# Effects of Nanofiber Scaffolds Coated with Nanoparticulate and Microparticulate Freeze Dried Bone Allograft on the Morphology, Adhesion, and Proliferation of Human Mesenchymal Stem Cells

**DOI:** 10.52547/ibj.26.3.193

**Published:** 2022-04-27

**Authors:** Shabnam Aghayan, Ehsan Seyedjafari, Shadi Hamidi

**Affiliations:** 1 Department of Periodontology, Faculty of Dentistry, Tehran Medical Sciences, Islamic Azad University, Tehran, Iran;; 2 Department of Biotechnology, College of Sciences, University of Tehran, Tehran, Iran;; 3 Dentist, Private Practice, Tehran, Iran

**Keywords:** Allografts, Bone regeneration, Mesenchymal stem cells, Nanofibers

## Abstract

**Background:**

Freeze dried bone allograft nanoparticles on a nanofiber membrane may serve as an ideal scaffold for bone regeneration. This study aimed to assess the biological behavior of human MSCs in terms of proliferation and adhesion to nanoparticulate and microparticulate FDBA scaffolds on PLLA nanofiber membrane.

**Methods:**

In this experimental study, PLLA nanofiber scaffolds were synthesized by the electrospinning method. The FDBA nanoparticles were synthesized mechanically. The FDBA nanoparticles and microparticles were loaded on the surface of PLLA nanofiber membrane. A total of 64 scaffold samples in four groups of n-FDBA/PLLA, FDBA/PLLA, PLLA and control were placed in 24-well polystyrene tissue culture plates; 16 wells were allocated to each group. Data were analyzed using one-way ANOVA and Bonferroni test.

**Results:**

The proliferation rate of MSCs was significantly higher in the nanoparticulate group compared to the microparticulate group at five days (*p* = 0.034). Assessment of cell morphology at 24 hours revealed spindle-shaped cells with a higher number of appendages in the nanoparticulate group compared to other groups.

**Conclusion:**

MSCs on n-FDBA/PLLA scaffold were morphologically more active and flatter with a higher number of cellular appendages, as compared to FDBA/PLLA. It seems that the nanoparticulate scaffold is superior to the microparticulate scaffold in terms of proliferation, attachment, and morphology of MSCs *in vitro*.

## INTRODUCTION

Bone defects caused by periodontal disease are reconstructed using autogenous bone grafts or synthetic biomaterials. Several types of biomaterials, including allografts, xenografts, and alloplasts, are available for this purpose. Allografts are among the commonly used bone substitutes for the reconstruction of bone defects and are available in two forms of FDBAs and DFDBAs^[1]^. At present, allografts are synthesized in the form of microparticles and are not accessible in nanoparticulate form. However, an earlier study on synthetic nanoparticulate bone substitutes has confirmed their superior efficacy for bone regeneration^[2]^.

Nanofiber scaffolds provide a suitable matrix for cell adhesion, proliferation, and differentiation and also play a fundamental role in tissue engineering^[3-7]^. An ideal scaffold should be able to perfectly mimic the biological structure and behavior of extracellular matrix^[7-9]^. Preliminary *in vitro* studies have demonstrated that stem cells well proliferate on nanofiber scaffolds^[10]^. 

 PLLA is a biodegradable and biocompatible polyester belonging to the few polymers approved for biomedical applications. It has been demonstrated that PLLA can be applied for the fabrication of tissue engineered scaffold and medical devices, as well as for commercial applications in the field of biomedical engineering^[11]^. PLLA can easily be processed into scaffold using different methods, such as electrospinning and three-dimensional printing. It can also be easily treated and coated with bioactive materials. The cost of its synthesis and production is lower than other similar polymers such as PGA and PLGA^[12]^. Therefore, in the present study, we used PLLA as a biomaterial for fabrication of nanofibrous scaffolds.

Stem cells are known for their unique self-renewal capacity. By generating ancestral cells, they provide an unlimited source of differentiated cells^[13]^. Recently, greater attention has been directed to adipose tissue among the adult stem cells because the adipose tissue has a higher number of adult stem cells, and adipocytes can be extracted in large amounts with minimal morbidity and mortality. These cells serve as a suitable source of mesenchymal cells with multi-potential differentiation capacity^[14-16]^. 

The current study aimed to compare the effect of nanofiber membranes coated with nanoparticulate and microparticulate FDBA on the morphology, adhesion, and proliferation of human MSCs. While there is extensive research on the applications of FDBA on bone restoration, to our knowledge, the nanophase FDBA has rarely been studied. This study is one of the first that has transformed the FDBA to a nanoversion and investigates its application as an alternative for bone restoration *in vitro*. Nanophase FDBA, as the present study proved for the first time, represents novel material formulations that enhance interactions and functions of MSCs. For this reason, nanophase FDBA clearly indicates a unique class of material formulations that promise enhanced bonding of orthopaedic/dental implants to bone, thus improving overall implant function.

## MATERIALS AND METHODS

**Preparation of nanofiber scaffolds**

In this *in vitro* experimental study, nanofiber scaffolds were synthesized by the electrospinning method. For this purpose, 12% PLLA (Sigma Aldrich) in dichloromethane (Merck, Germany) was transferred to a 5-mL syringe with a 21-gauge needle. A stainless steel collector was placed at 15 cm distance from the needle in order to collect the electrospinning nanofibers. The solution was fed into the needle via a tube at a rate of 1 mL/h. Next, 20 kV voltage was induced between the needle and collector. By doing so, the solution left the needle and accumulated in the collector in the form of very thin threads of fiber. After achieving 200 µm of thickness, the mat was separated from the collector and placed in vacuum in order for the solvent to evaporate. The oxygen plasma treatment was performed to modify the surface with lower than 44 Hz frequency in a quartz cylindrical container. Pure oxygen was generated in a reaction chamber at 0.04 mbar pressure and was then charged for five minutes. 

**Preparation of nanoparticulate bone substitute**

**Fig. 1:**
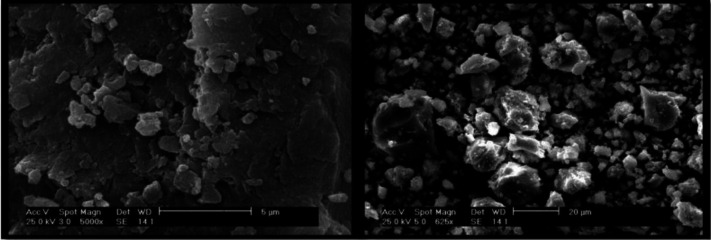
SEM micrographs of nano bone powder (the size of FDBA particles ranged from 100 nm to 10 µm).

A mechanical ball mill was used to synthesize nanoparticles from microparticles. The entire system was sterilized with alcohol. In this process, 120-g stainless steel balls were used to grind 6 g of bone substitute. In other words, the weight ratio of stainless steel balls to bone substitute was 20:1. The ball mill operated at 325 rpm for six hours^[17]^. Two containers containing the balls and powder were present. Using the ball mill, the bone substitute powder was ground. The SEM assessment confirmed the synthesis of submicron (nanometer-scale) bone substitute. In this study, we tried our best to decrease the size of microparticles and create nanoparticles. The aim was to generate particles with the size of >10 nm because smaller nanoparticles would pass through the cell membrane and the endothelial lining of blood vessels and enter the blood stream, which would result in complications. Moreover, over-grinding of particles of would prevent their homogenous mixing with micron-scale particles and would enhance their wash-out with a small amount of blood or any other fluid due to their very low weight. Therefore, in this study, the size of particles ranged from 100 nm to 10 µm. Very small amounts of 20-30 µm particles were also present, which were disregarded due to their very small percentage. The primary microparticles ranged in size from 150 to 1000 µm (Fig. 1). The synthesized bone substitute powder with optimal physical properties had 25 wt% nanoparticles to the total weight percentage of the powder. In this ratio, a homogenous distribution nanoparticles on the surface of FDBA microparticles was achieved. High amounts of nanoparticulate and microparticulate powders could not be well mixed. Thus, each time, 0.25 g of nanopowder was mixed with 0.75 g of micropowder such that it reached a total weight of 1 g, which was poured into a glass screw top vial and was shaken on a shaker for 20 minutes. This process was repeated for each 1 g of powder in order to be reproducible. If the amount of nanoparticle was higher or lower, the mixture would not be homogeneous and if shaken, the nanoparticles would be separated from the microparticles. Therefore, it should be shaken prior to use as nanoparticles and microparticles may be separated during transfer of vials and over time. In this study, the vial was mixed before use. It should be noted that the 25% ratio is highly important to achieve the desired consistency^[17]^.

**Gamma sterilization**

 As stated in ISO 11137 (https://www.iso. org/sites/directives/current/consolidated/index.xhtml), sterilization of medical equipment can be carried out at a dose of 15 kGy or 25 kGy. Due to easy access and low costs of gamma radiation and its low effect on mechanical properties of long bones, in comparison to other means of sterilization, this method is more acceptable and widely used^[18,19]^. Therefore, the sterilization of bone substitutes was performed in Hamanand Saz Baft Kish Company (Kish, Iran) according to the AATB and FDA guidelines. All the equipment and services, including the clean rooms, were in conformity with the cGMP. All tissues were prepared in a controlled environment known as class 1000 clean room and processed in a sterile environment (class 10-1000). After synthesizing the nanoparticles, gamma sterilization was performed based on the protocol such that a minimum of 25 GY was absorbed by the tissue^[20]^. According to ISO 10993-5, the processed bone substitutes were nontoxic. The final product was quality-controlled and supplied. 

**Loading of n-FDBA on the surface of PLLA nanofiber membranes**

n-FDBA solution (1%) was dissolved in deionized water and placed in an ultrasonic bath for 20 minutes. In order to load n-FDBA on the surface of PLLA nanofiber membranes, the plasma-modified scaffold was immersed in n-FDBA aqueous solution overnight and was then rinsed with deionized water and dried under vacuum. The same process was employed for loading microparticulate FDBA on the surface of nanofiber membranes^[21,22]^. Pristine PLLA nanofibers revealed a substantially low capacity for cell attachment as a result of the high hydrophobicity. Therefore, plasma-treated PLLA was used in all experiments and referred to as PLLA in this study^[23]^.

**ATR-FTIR spectroscopy**

The coating of FDBA on the surface of PLLA nanofibers was investigated by FTIR-ATR. The spectra were recorded using an Equinox 55 spectrometer (Bruker Optics, Germany) equipped with a DTGS detector and a diamond ATR crystal.

**Contact angle measurement**

The water contact angle of the surface of nanofibers before and after surface treatment and FDBA coating was evaluated by the sessile drop method with a G10 contact angle goniometer (Kruss, Germany) at room temperature. A water droplet was placed on the scaffold surface, and the contact angle was measured after 10 s.

**Culture of human adipose tissue MSCs**

Adipose tissue is the most common and perhaps the simplest way to harvest MSCs^[24,25]^. In addition, at the same two-dimensional culture condition, AT-MSCs exhibited higher proliferation and osteogenic differentiation capacities compared to BM-MSCs^[26,27]^. The stem cells were cultured in DMEM, supplemented with 30% FBS, dexamethasone (100 nM), penicillin (100 U/ml), streptomycin (0.1 mg/ml), and L-glutamine (2 mM), all from Gibco (Grand Island, NY, USA) except for dexamethasone (Sigma Aldrich). After two weeks, the cells were detached with 0.25% Trypsin-EDTA (Gibco) and resuspended in DMEM supplemented with 10% FBS. The cells were cultured in a humidified atmosphere at 37 °C and 5% CO_2_, and after 24 hours, unattached cells were rinsed off with phosphate buffered saline^[28]^. For cell culture, round-shaped scaffolds with 1.5 cm diameter were cut and placed in 24-well polystyrene cell culture plates. Following sterilization by 70% ethanol, the scaffolds were placed in a basic culture medium overnight to enhance cell adhesion. A total of 10^4^ cells were immersed in 200 µL of culture medium in each well. The cells were cultivated on four types of scaffolds, namely the TCP as the control group (that only contained MSCs), FDBA/PLLA, PLLA, and n-FDBA/PLLA, and incubated for 30 minutes^[23]^.

**Assessment of adhesion and morphology of the cells**

The morphology of adipose tissue MSCs on all scaffolds was inspected under a SEM one day after culture. For the assessment of cell morphology, the cells were seeded onto the scaffolds and incubated in the aforementioned normal conditions for 24 hours. The cells were then rinsed twice with phosphate buffered saline, fixed in 2.5% glutaraldehyde (Sigma Aldrich) at room temperature for one hour, rinsed, and dehydrated using different concentrations of ethanol. The samples were then gold-coated and inspected under a SEM (Nikon, Japan) at ×1000 magnification. The surface area of the scaffold covered with cells in square micrometers, and the sphericity of cells (ratio of the smaller diameter to larger diameter of each cell; 0 indicated more elongated cells and 1 indicated more spherical cells) were evaluated^[29]^. To assess the cell adhesion, the MTT assay (Sigma Aldrich) was applied in the first 24 hours after cell culture on scaffolds. Briefly, nanofiber membranes were sterilized and placed in a 24-well culture plate containing 10^4^ cells/cm_2_ and incubated at 37 °C and 5% CO_2_. After 1, 3,5 and 7 days of culture, 50 µL of the MTT solution was added to each well and incubated at 37 °C for 3.5 hours. Next, 1 mL of dimethyl sulfoxide (Merck) was added to the solution to break down intracellular formazan crystals. Thereafter, the OD was measured by a spectrophotometer (BioTek Instruments, USA) at 570 nm wavelength^[23,29]^. 

**Statistical analysis**

Data were analyzed using SPSS version 20 via one-way ANOVA and Bonferroni test. In our case, there are four groups of control (TCP), FDBA/PLLA, PLLA, and n-FDBA/PLLA. ANOVA test analyzes the levels of variance within the groups through samples taken from each of them and compares their mean to determines whether the differences between groups of data are statistically significant^[30]^.

## RESULTS

This study assessed the morphology, adhesion, and proliferation of MSCs on 64 scaffold samples in four groups of control (TCP), FDBA/PLLA, PLLA, and n-FDBA/PLLA. Sixteen samples were evaluated in each group, and SEM and MTT assessments were performed at 1, 3, and 5 days. 

As shown in Table 1, ANOVA revealed a significant difference among the four groups at each time point (*p* < 0.05). Thus, pairwise comparisons of the groups were carried out using Bonferroni post-hoc test at each time point (Tables 2-4). Based on Table 2, on day 1, cell adhesion in the TCP group was significantly different from that in the three other groups (*p* < 0.05). No other significant differences were noted in pairwise comparisons (*p* > 0.05). On day three, cell proliferation in TCP group was significantly different from that of the remaining three groups (*p* < 0.05; Table 3); other pairwise comparisons did not display any significant difference in cell adhesion (*p* > 0.05). As depicted in Table 4, on day 5, cell proliferation in the TCP group had a significant difference with that in FDBA/PLLA group (*p* < 0.05). Also, cell proliferation in n-FDBA/ PLLA group had a significant difference with that in FDBA/PLLA group (*p *< 0.05) such that cell proliferation in n-FDBA/PLLA group was higher than that in FDBA/PLLA group. 

**Fig. 2:**
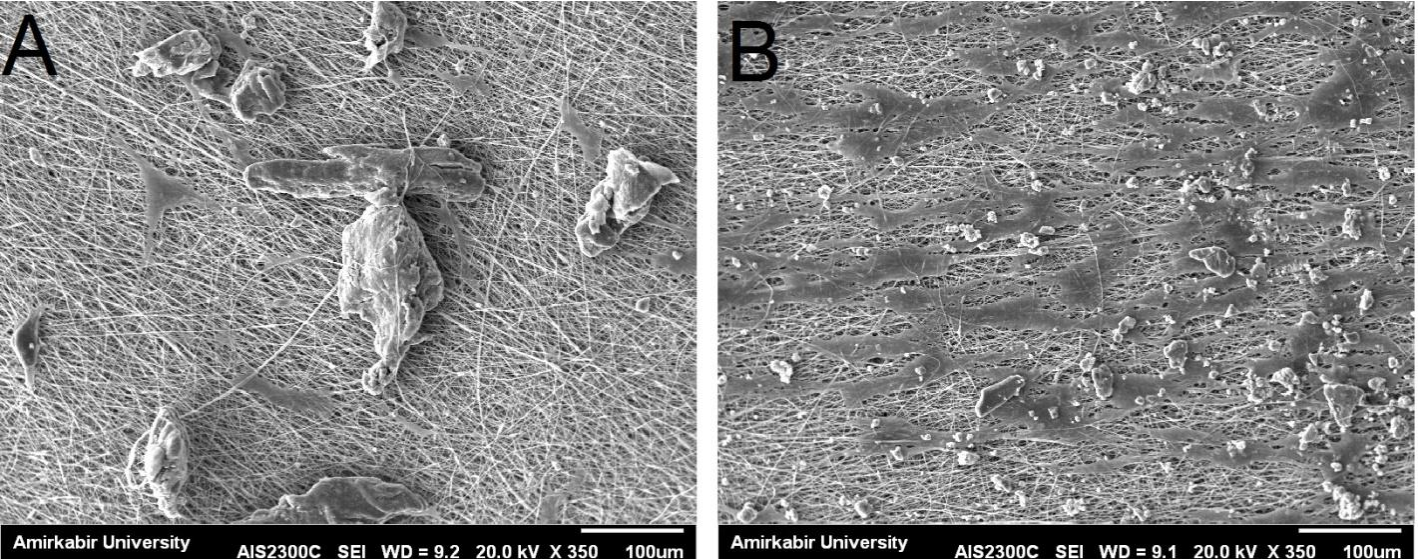
SEM micrograph of mesenchymal stem cells coated with (A) FDBA/PLLA and (B) nFDBA/PLLA. As shown in both images, there is a significantly higher number of MSCs on n-FDBA/PLLA compared to FDBA/PLLA. The MSCs on n-FDBA/PLLA scaffold were morphologically more active and flatter with higher number of cellular appendages compared to FDBA/PLLA.

Morphological assessment of cells using SEM on day one revealed the presence of the higher number of cell appendages, and their spindle shape in the nanoparticulate group compared with other groups, indicating that they were biologically active (Fig. 2).

**Table 1:** Results of ANOVA for cell adhesion on day 1 and cell proliferation on days 3 and 5

		**Group**			
		**TCPs**	**PLLA**	**n-FDBA/PLLA**	**FDBA/PLLA**
Adhesion (day 1)	**Mean ± SD**	0.275 ± 0.016	0.242 ± 0.011	0.228 ± 0.019	0.243 ± 0.020
	*p* value (ANOVA)	0.003			
Proliferation (day 3)	Mean ± SD	0.492 ± 0.018	0.306 ± 0.018	0.358 ± 0.024	0.307 ± 0.043
	*p *value (ANOVA)	0.0001			
		
Proliferation (day 5)	Mean ± SD	1.092 ± 0.071	1.004 ± 0.036	1.058 ± 0.041	0.937 ± 0.079
*p* value (ANOVA)	0.005			

**Table 2:** Pairwise comparisons of the groups in terms of cell adhesion on day 1 using the Bonferroni test

**Group (I)**	**Group (J)**	**Mean Difference (I-J)**	**Std. Error**	***p*** ** value**
TCPs	PLLA	0.033	0.011	0.042
	n-FDBA/PLLA	0.047	0.011	0.003
	FDBA/PLLA	0.032	0.011	0.045
PLLA	n-FDBA/PLLA	0.014	0.011	1.000
	FDBA/PLLA	0.000	0.011	1.000
n-FDBA/PLLA	FDBA/PLLA	-0.014	0.011	1.000

**Table 3:** Pairwise comparisons of groups in terms of cell adhesion on day 3 using the Bonferroni test

**Group (I)**	**Group (J)**	**Mean Difference (I-J)**	**Std. Error**	***p*** ** value**
TCPs	PLLA	0.186	0.018	0.000
	n-FDBA/PLLA	0.134	0.018	0.000
	FDBA/PLLA	0.186	0.018	0.000
PLLA	n-FDBA/PLLA	-0.052	0.018	0.055
	FDBA/PLLA	0.000	0.018	1.000
n-FDBA/PLLA	FDBA/PLLA	0.052	0.018	0.057

**Table 4:** Pairwise comparisons of cell proliferation in the groups on day 5

**Group (I)**	**Group (J)**	**Mean Difference (I-J)**	**Std. Error**	***p*** ** value**
TCPs	PLLA	0.087	0.038	0.210
	n-FDBA/PLLA	0.034	0.038	1.000
	FDBA/PLLA	0.155	0.038	0.005
PLLA	n-FDBA/PLLA	-0.054	0.038	1.000
	FDBA/PLLA	0.067	0.038	0.572
n-FDBA/PLLA	FDBA/PLLA	0.121	0.038	0.034

**Fig. 3:**
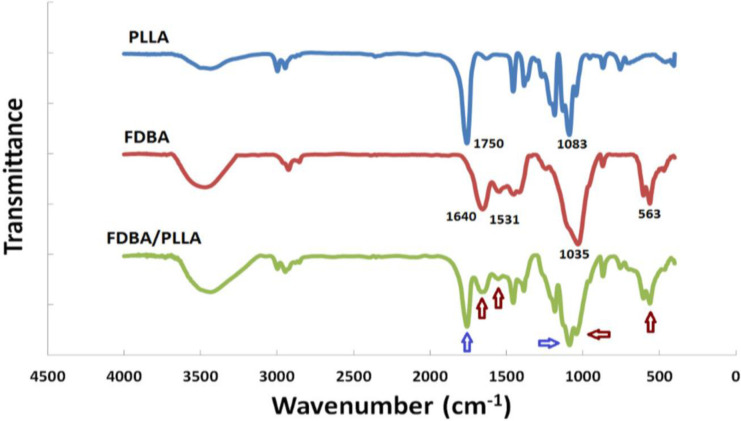
The presence of FDBA on the surface of PLLA scaffolds conﬁrmed via ATR-FTIR.

Contact angle measurements showed that PLLA scaffolds became completely hydrophilic after plasma treatment, and FDBA coating did not affect its hydrophilicity. The presence of FDBA on the surface of PLLA scaffolds was conﬁrmed via ATR-FTIR (Fig. 3). Strong characteristic peaks of PLLA was detected at 1750 cm^-1^ for C=O group and at 1083 cm^-1^ for C-O stretching. Peaks at 563 and 1035 cm^-1^ are referred to the vibrations in PO_4_^3-^, which is in the chemical structure of hydroxyapatite embedded in FDBA. Existence of FDBA was also affirmed through the Amide I and II bands of its proteins, which were detected at 1640 and 1531 cm^-1^, respectively.

## DISCUSSION

This study evaluated the morphology, adhesion, and proliferation of MSCs on 64 scaffold samples in four groups of control (TCP), FDBA/PLLA, PLLA, and n-FDBA/PLLA. FDBA/PLLA and n-FDBA/PLLA are composed of PLLA nanofiber membranes with thin threads and numerous pores. This structure mimics the extracellular matrix and regulates cell adhesion and proliferation^[31,32]^. 

Assessment of cell proliferation using the MTT assay at 3 and 5 days after cell culture on the surface of scaffolds revealed that cell proliferation in all four groups increased with time. On days 3 and 5, cell proliferation in the TCP group was significantly higher than that in the other three groups. At the end of day 5, cell proliferation in n-FDBA/PLLA group was significantly higher than that in FDBA/PLLA group. 

Shakir *et al.*^[33]^ reported the results of the MTT assay at 24 hours after cell culture and implied that cell viability in the nano-hydroxyapatite combined with chitosan and polysaccharide group was higher than that in other groups. Ramezanifard *et al.*^[29]^ showed that in terms of cell morphology, MSCs had a similar spindle-shaped morphology and optimal adhesion to the surface of nanofiber membranes in all scaffold groups. In terms of cell proliferation, a significant increase in cell proliferation was noted in all groups. Nonetheless, cell proliferation decreased after five days because the cells showed signs of induction and differentiation to osteogenic cells. From day five to day seven, mesenchymal cells in the control group showed higher proliferation than those in the nanofiber scaffold groups. In our study, at the end of day five, the control group showed higher proliferation rate than other groups. In general, higher cell proliferation in the control group at each time point can be due to the smoothness of the plate surface in the control group compared to the rough surface of other scaffolds. Surface topography affects the adhesion and differentiation of cells; however, its effect depends on the cell type. Different cells display different behaviors on smooth and porous surfaces such that rougher surfaces are more suitable for osteoblasts, and smoother surfaces are more suitable for fibroblasts and mesenchymal cells^[34,35]^. Also, the TCP (control group) 

is an ideal scaffold for cell proliferation; however, it cannot be directly used in the clinical setting^[35]^. Hayrapetyan *et al.*^[27]^ assessed cell proliferation and differentiation by evaluating the cellular DNA content, alkaline phosphatase activity, and calcium deposition on days 7 and 14, which were different from the assessment tool used in our study (since we performed the MTT assay). 

In terms of cellular behavior, adipose tissue MSCs exhibited higher proliferation, differentiation, and mineralization capacity in nanohydroxyapatite/collagen structures compared to the bone marrow MSCs. The high proliferation rate of adipose tissue MSCs indicates their potentially high cell interactions compared to the bone marrow MSCs. Greater proliferation and differentiation were noted in the nanoparticle group compared to the microparticle group, which was in line with our study. Gandhimathi *et al.*^[36]^ have disclosed that cell proliferation is significantly higher on nanoparticulate scaffolds compared to other scaffolds on days 14 and 21. In these two days, cell proliferation on the nanoparticulate scaffold started to decrease because of the initiation of MSCs to differentiate to osteogenic cells. However, in our study, the proliferation rate of cells increased during the aforementioned five days. The MSCs seeded on the nanoparticulate scaffold had a cubic shape and higher number of cell appendages, which was in agreement with our observations.

Our results confirmed the findings of previous studies showing that n-FDBA/PLLA group significantly outperformed in terms of proliferation, adhesion, and morphology of MSCs *in vitro*^[37-39]^. According to the existing literature, application of nanoparticles offers a superior environment for protein adsorption and cellular interactions^[40]^. In other words, nanostructured scaffolds have several distinctive surface properties, such as higher surface area, superior mechanical, electrical, optical, or magnetic properties. These surface characteristics would increase protein adherence and could result in improvement in cell attachment compared to other (control) groups. Furthermore, nanomaterials surfaces present a relatively higher nanoscale roughness and specific surface chemistries, wettability, and surface energies^[41]^. 

In our study, superior biological behavior of cells in the nanoparticle group compared to the microparticle group may be attributed to the fact that nanoparticles, due to their very small size, can stimulate the cell surface receptors and activate cell signaling, causing cell proliferation and osteogenesis. Under *in vitro* conditions, n-FDBA/PLLA and FDBA/PLLA scaffolds showed similar behavior in terms of adhesion of MSCs. However, the proliferation rate of MSCs was significantly higher on n-FDBA/PLLA compared to FDBA/PLLA scaffold on day five. The MSCs on n-FDBA/PLLA scaffold were more active and had a wider morphology with more cellular appendages. It seems that the nanoparticulate scaffold is more suitable than the microparticulate scaffold in terms of proliferation, adhesion, and morphology of MSCs *in vitro*.

## DECLARATIONS

### Ethical statement

Not applicable.

### Data availability

The analyzed data sets generated during the study are available from the corresponding author on reasonable request.

### Author contributions

SG: concept and design, analysis and interpretation of data, drafting of the manuscript, and critical revision of the manuscript for important intellectual content; SH: analysis and interpretation of data and statistical analysis; ES, critical revision of the manuscript for important intellectual content.

### Conflict of interest

None declared.

### Funding/support

This research did not receive any specific grant from funding agencies in the public, commercial, or not-for-profit sectors.
